# Differences in hormonal levels between heterozygous *CYP21A2* pathogenic variant carriers, non-carriers, and females with non-classic congenital hyperplasia

**DOI:** 10.20945/2359-3997000000437

**Published:** 2022-03-16

**Authors:** Rita Santos Silva, Berta Carvalho, Jorge Pedro, Cíntia Castro-Correia, Davide Carvalho, Filipa Carvalho, Manuel Fontoura

**Affiliations:** 1 Universidade do Porto Faculdade de Medicina Centro Hospitalar Universitário S. João Portugal Departamento de Endocrinologia Pediátrica, Centro Hospitalar Universitário S. João; Faculdade de Medicina da Universidade do Porto, Portugal; 2 Universidade do Porto Faculdade de Medicina Departamento de Patologia Portugal Genética, Departamento de Patologia, Faculdade de Medicina da Universidade do Porto, Portugal.; 3 Universidade do Porto Instituto de Investigação e Inovação em Saúde Faculdade de Medicina Portugal Departamento de Endocrinologia, Diabetes e Metabolismo, Centro Hospitalar Universitário S. João; Faculdade de Medicina, Instituto de Investigação e Inovação em Saúde, Universidade do Porto, Portugal

**Keywords:** Heterozygous carrier, 17-hydroxyprogesterone, precocious pubarche, hyperandrogenism

## Abstract

**Objective:**

CYP21A2 mutation heterozygote carriers seem to have an increased risk of hyperandrogenism. However, the clinical relevance of the heterozygote carrier status and the reliability of hormonal testing in discriminating a carrier from a non-carrier are puzzling questions. We aimed to characterize a population of Portuguese females suspected of having non-classic congenital adrenal hyperplasia (NC-CAH) due to clinical and biochemical criteria and who have undergone CYP21A2 molecular analysis.

**Subjects and methods:**

Retrospectively, we have analyzed the clinical records of 131 females (32 girls aged 3-9 and 99 adolescents and premenopausal women aged 13-49) who underwent complete CYP21A2 molecular analysis due to suspicion of NC-CAH. We divided included participants into three groups according to the CYP21A2 molecular analysis: NC-CAH females (46), heterozygous carriers (49), and wild type (36). We then compared clinical signs and symptoms as well as biochemical and molecular data between carriers and NC-CAH individuals and between carriers and wild type females. We measured 17OHP by electrochemiluminescence immunoassay.

**Results:**

Clinical features were similar between groups. Heterozygous carriers presented higher basal and post-cosyntropin 17-hydroxyprogesterone (17OHP) than wild type individuals (p < 0.05) and lower basal and stimulated 17OHP levels than NC-CAH patients (p < 0.05). We discovered a considerable overlap between 17OHP levels among groups. The most common pathogenic variant we identified was p.Val282Leu.

**Conclusion:**

In this population of hyperandrogenic women and children, heterozygous carriers showed higher basal and stimulated 17OHP than non-carriers although normal basal and stimulated 17OHP responses do not exclude heterozygosity for CYP21A2 pathogenic variants. In this study, only the molecular analysis presented good sensitivity in identifying heterozygotes.

## INTRODUCTION

Congenital adrenal hyperplasia (CAH) entails a group of inborn errors of adrenal steroidogenesis. Deficiency of 21-hydroxylase (21OHD) accounts for 95% of the CAH cases. It results in defective conversion of 17-hydroxyprogesterone (17OHP) to 11-deoxycortisol and leads to hypocortisolism and hyperandrogenism due to the accumulation of precursor steroid hormones. It has a broad spectrum of clinical forms, ranging from classic salt wasting, classic simple virilizing and non-classic (NC). The NC female patients generally present premature pubarche in midchildhood or hirsutism, severe acne, oligomenorrhea, or infertility in adolescence and adulthood.

Non-classic congenital adrenal hyperplasia (NC-CAH) affects around 1 in 100 to 1 in 1,000 individuals ( 1 ), and the frequency of heterozygote carriers is approximately 1 in 55 in the European population ( 2 ).

Pathogenic variants in the *CYP21A2* gene cause various degrees of dysfunction in enzymatic activity, and a strong genotype-phenotype correlation emerges, especially in the disease’s most severe forms ( 3 ). Genetically, CAH is an autosomal recessive disorder; therefore, an affected individual must present with at least two biallellic pathogenic variants, and the phenotype is conferred by the less deleterious variant ( 4 ).

The clinical relevance of the heterozygote carrier *status* and the reliability of hormonal testing in discriminating a carrier from a non-carrier are still puzzling questions. A heterozygous carrier’s normal allele should encode a CYP21A2 protein with almost complete activity, which ensures a normal physiological function. However, heterozygous carriers of *CYP21A2* pathogenic variants seem to have an increased risk of hyperandrogenism, which may manifest as premature pubarche, accelerated growth, apocrine body odor, and seborrhea in childhood ( 5 – 8 ) and hirsutism, oligomenorrhoea, acne, and infertility in adolescence and adulthood ( 9 – 12 ).

In cohorts of hyperandrogenic women and children, the frequency of the carrier *status* varies based on various inclusion criteria and ranges from 8% to 35% ( 7 , 12 – 15 ). Although some studies have shown a higher prevalence of *CYP21A2* carriers in hyperandrogenic individuals than in the general population ( 16 , 17 ), others have shown no such difference ( 13 , 18 – 20 ).

We aimed to characterize a population of 131 Portuguese females, aged 3-49, suspected of having NC-CAH due to clinical and biochemical criteria, who have undergone *CYP21A2* molecular analysis. In this population of 131 females, we aimed to compare 49 heterozygous carriers of *CYP21A2* pathogenic variants with 46 NC-CAH individuals (2 pathogenic variants identified) and 36 subjects without any pathogenic variant on *CYP21A2* .

## SUBJECTS AND METHODS

### Population

We have analyzed a population of 131 females referred to our hospital’s endocrinology and pediatric endocrinology clinics in the past 10 years who underwent *CYP21A2* molecular analysis due to suspected NC-CAH. This population included 32 (24%) girls aged 3-9 and referred to our clinics due to precocious pubarche as well as 99 (76%) adolescents and premenopausal women aged 13-49 referred due to hirsutism, oligomenorrhea, severe acne, alopecia, and/or infertility.

We performed *CYP21A2* molecular analysis for all these participants. Criteria for the molecular analysis in our center were basal 17 OHP > 2 ng/mL and post-ACTH > 10 ng/mL, familial history of CAH plus symptoms of hyperandrogenism, or severe hyperandrogenic signs or symptoms.

Participants fulfilled clinical and biochemical criteria, and we divided them into three groups according to the *CYP21A2* molecular analysis. Group I included 46 females (12 girls, 34 women) with a diagnosis of NC-CAH, which was defined as normal genitalia at birth and clinical signs of hyperandrogenism that appeared later in childhood (pubarche, overgrowth, apocrine body odor) and adolescence (hirsutism, acne, oligomenorrhea, infertility), together with a genetic analysis showing two pathogenic variants in both *CYP21A2* alleles (homozygous or compound heterozygous). Group II included 49 heterozygous carriers of the *CYP21A2* pathogenic variant (10 girls, 39 women) with clinical features overlapping NC-CAH but having a single disease-causing CAH *CYP21A2* pathogenic variant in one allele. Group III included 36 females (10 girls, 26 women) with features overlapping NC-CAH but without any *CYP21A2* pathogenic variant identified.

### Clinical assessment

An endocrinologist or a pediatrician evaluated all patients enrolled in the study at referral, and clinical signs and symptoms were abstracted from clinical records. Premature pubarche was defined as the development of pubic hair before the age of 8 in girls and 9 in boys ( 21 ). Hirsutism was defined as a Ferriman-Gallwey score ≥8 ( 22 ) and oligomenorrhea as menstrual cycles lasting 35-90 days.

### Biochemical analysis

We measured dehydroepiandrosterone sulphate (DHEAS), 17OHP, androstenedione, and total testosterone by electrochemiluminescence immunoassay on the Elecsys 2010 Modular Analytics E170 analyzer (Roche Diagnostics). In post-pubertal women, we conducted the analyses in the follicular phase, before 9 am. We performed the stimulation test with administration of 250 µg synthetic cosyntropin (Synachten^®^) and measured 17OHP at 0 and at 60 min post-cosyntropin.

### Molecular analysis

We selectively amplified functional *CYP21A2* gene sequencing by polymerase chain reaction (PCR) into two partially overlapping fragments, avoiding the co-amplification of the pseudogene *CYP21A1P* ( 23 , 24 ). Using the complete gene sequencing strategy, on the promoter region, approximately 130bp upstream, we sequenced the start codon by Sanger sequencing. We sequenced all coding sequences and intron-exon boundaries with internal primers that covered the entire *CYP21A2* gene. We performed both restriction fragment length polymorphism (RFLP) for large deletion/conversions involving the *CYP21A2* promoter region and MLPA for detection of large rearrangements ( 23 , 24 ). In all cases where it was mandatory, we performed parental segregation studies to determine whether the variants were in cis or in trans.

We determined nomenclature of *CYP21A2* variants according to the Ensembl transcript *CYP21A2* -002: ENST00000418967.6 (NM_000500.7 from *National Center for Biotechnology Information* ).

### Statistical analysis

We performed statistical analysis using SPSS^®^ v.20. We applied nonparametric tests to compare independent samples and used chi-square tests to determine the association between categorical variables. We established comparisons between Group II (carriers) and Group I (NC-CAH patients) and between Group II (carriers) and Group III (wild type individuals). We regarded P values below 0.05 as statistically significant.

### Ethics approval

We conducted all procedures in this study in accordance with the national committee for data protection and with the 1964 Helsinki declaration or comparable ethical standards. This original study had the approval from the Ethical Committee, Centro Hospitalar Universitário S. João, Porto, Portugal. We obtained informed consent from the participants.

## RESULTS

Table 1 summarizes the 131 included females’ ages at referral, clinical phenotypes at presentation, genotypes, and biochemical data.

**Table 1:** Comparison between age at referral, phenotype at presentation, genotype, and biochemical data of the 131 included females, by age and genotype groups

		All	Group I (+/+)	Group II (+/−)	Group III (−/−)	p
All						
Number of individuals, n (%)		131 (100%)	46 (35%)	49 (37%)	36 (28%)	
Age at referral, years (median, min-max)		22.6 (3.3-49.6)	23.0 (5.1-49.6)	22.6 (3.3-48.3)	22.5 (3.7-46.0)	NS †
Childhood hyperandrogenism*						
Number of individuals (%)		32 (100%)	12 (38%)	10 (31%)	10 (31%)	
Age at referral, years (median, min-max)		6.3 (3.3-9.2)	6.9 (5.1-9.6)	5.5 (3.3-7.3)	6.6 (3.7-8.2)	NS †
Phenotype at presentation (%)						
	Premature pubarche	97%	92%	100%	100%	NS ▼
	Tall stature	3%	8%			
Genotype (number of individuals)						
	p.Val282Leu/p.Val282Leu		7 (58%)			
	p.Val282Leu/del/conv promotor CYP21A2		2 (17%)			
	p.Val282Leu/p.Gly111ValfsTer21		1 (8%)			
	p.Val282Leu/p.Pro454Ser		1 (8%)			
	p.Val282Leu/p.R444X		1 (8%)			
	p.Val282Leu/-			9(90%)		
	c.290-13A/C>G/-			1(10%)		
Biochemical data						
Basal 17OHP, ng/mL (median, min-max)		4.8 (0.4-54.3)	16.7 (1.4-54.3)	3.3 (0.9-9.0)	2.0 (0.4-1.3)	**<0.05**†
Pos-ACTH 17OHP, ng/mL (median, min-max)		12.5 (4.4-79.0)	55.1 (17.3-79.0)	11.9 (6.2-13.9)	7.7 (4.4-11.1)	**<0.05**†
DHEAS, µg/dL (median, min-max)		86.3 (40.0-178.2)	125.4 (46.0-178.2)	77.5 (46.9-141.8)	74.2 (40.0-150.3)	NS †
Androstenedione, ng/mL (median, min-max)		1.0 (0.0-2.9)	1.5 (0.8-2.9)	0.5 (0.0-1.8)	0.4 (0.03-1.2)	NS †
Total testosterone, ng/mL (median, min-max)		0.1 (0.0-0.3)	0.1 (0.1-0.3)	0.1 (0.1-0.2)	0.0 (0.0-0.2)	NS †
Adolescence or adulthood hyperandrogenism**						
Number of individuals (%)		99 (100%)	34 (34%)	39 (39%)	26 (26%)	
Age at referral, years (median, min-max)		27.9 (13.2-49.6)	28.2 (13.2-49.6)	27.3 (14.2-48.3)	27.7 (13.5-46.0)	NS †
Phenotype at presentation (%)						
	Hirsutism	76%	63%	91%	82%	NS ▼
	Oligomenorrhea	11%	19%	0%	9%	
	Severe acne	5%	6%	9%	0%	
	Alopecia	5%	6%	0%	9%	
	Infertility	3%	6%	0%	0%	
Genotype, n (%)						
	p.Val282Leu/p.Val282Leu		17(50%)			
	p.Val282Leu/del/conv promotor CYP21A2		17(50%)			
	p.Val282Leu/-			39(100%)		
Biochemical data						
Basal 17OHP, ng/mL (median, min-max)		3.4 (0.1-30.6)	7.4 (0.6-30.6)	3.7 (0.6-15.7)	1.7 (0.1-5.7)	**<0.05**†
Pos-ACTH 17OHP, ng/mL (median, min-max)		13.0 (2.7-21.9)	16.5 (13.0-21.9)	10.8 (4.2-13.0)	5.5 (2.7-9.5)	**<0.05**†
DHEAS, µg/dL (median, min-max)		198.2 (0.7-598.1)	209.3 (0.7-399.4)	138.2 (12.3-598.1)	204.8 (2.1-536.0)	NS †
Androstenedione, ng/mL (median, min-max)		3.2 (0.2-8.8)	3.8 (0.2-8.8)	2.7 (0.2-7.3)	2.2 (0.3-8.4)	NS †
Total testosterone, ng/mL (median, min-max)		0.4 (0.03-134.0)	0.5 (0.0-1.0)	0.6 (0.1-2.1)	0.3 (0.0-134.0)	NS †

Group I (+/+) included females with a diagnosis of NC-CAH (hyperandrogenism and genetic analysis showing 2 pathogenic variants in the *CYP21A2* ). Group II (+/−) included heterozygous carriers of *CYP21A2* pathogenic variant. Group III (−/−) included females with features overlapping NC-CAH but without any *CYP21A2* pathogenic variant identified.

* The “childhood hyperandrogenism” group includes 32 girls aged 3-9 years-old at referral due to precocious pubarche or tall stature.

** The “adolescence or adulthood hyperandrogenism” included 99 adolescents and older women aged 13-69 years at referral due to infertility, oligomenorrhea, severe acne, alopecia, and/or hirsutism.

† Non-parametric tests (Group II compared to I and III).

▼ Pearson Chi-square.

NS: non-significant (p > 0.05); 17OHP: 17-hydroxyprogesterone; DHEAS: dehydroepiandrosterone sulphate.

The participants’ median age at referral was 6.3 years in children and 27.9 years in adolescents and adults. We found no significant differences between groups.

Children presented mostly with premature pubarche (97%), and adolescents and adults presented with hirsutism (76%) and oligomenorrhea (11%). We found no significant differences in presentation between NC-CAH patients (Group I), heterozygous carriers (Group II), and wild type individuals (Group III).

We found statistically significant differences in basal and stimulated 17OHP levels in both the children and the adolescents/adult populations between carriers (Group II) and NC-CAH patients (Group I) and between carriers (Group II) and wild type subjects (Group III).

In children, heterozygous carriers showed higher basal and cosyntropin-stimulated 17OHP than wild type girls (basal 17OHP: median 3.3 *vs.* 2.0 ng/mL; p < 0.05 stimulated 17OHP: median 11.9 *vs.* 7.7 ng/mL; p < 0.05) and lower basal and stimulated 17OHP than NC-CAH patients (basal 17OHP: median 3.3 *vs.* 16.7 ng/mL; p < 0.05; stimulated 17OHP: median 11.9 *vs.* 55.1 ng/mL; p < 0.05, respectively).

In adolescents and adults, heterozygous carriers showed higher basal and cosyntropin-stimulated 17OHP than wild type women (basal 17OHP: median 3.7 *vs.* 1.7 ng/mL; p < 0.05; stimulated 17OHP: median 10.8 *vs.* 5.5 ng/mL; p < 0.05) and lower basal and stimulated 17OHP than NC-CAH patients (basal 17OHP: median 3.7 *vs.* 7.4 ng/mL; p < 0.05); stimulated 17OHP: median 10.8 *vs.* 16.5 ng/mL; p < 0.05).

**Figure 1:**
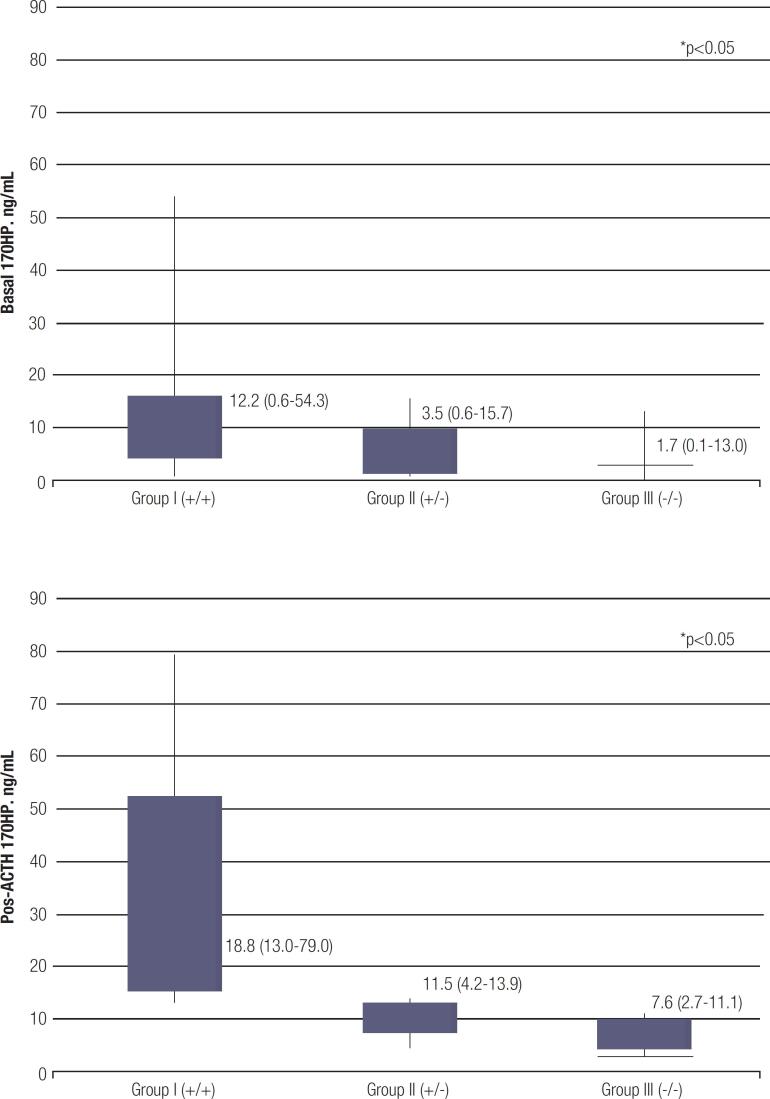
Basal (1A) and pos-ACTH (1B) 17OHP levels (ng/mL) according to groups (median (min-max)) . Group I (+/+) included females with NC-CAH (hyperandrogenism and genetic analysis showing 2 pathogenic variants in the *CYP21A2* ). Group II (+/−) included heterozygous carriers. Group III (−/−) included wild type females. *Nonparametric tests were applied to compare Group II (carriers) and Group I (NC-CAH patients), and Group II (carriers) and Group III (wild type individuals) (p < 0.001 for all the comparisons).

Figure 1 shows basal and post-ACTH 17OHP levels according to groups, for the whole sample. Differences in basal and pos-ACTH 17OHP levels between children and adolescent/adult women were non-significant (medians, respectively, 4.8 *vs.* 3.4 ng/mL (p = 0.278) and 12.5 *vs.* 13.0 ng/mL (p = 0.409).

We found no statistically significant differences in DHEAS, androstenedione, or total testosterone levels between groups in either children or adolescent and adult women.

Regarding genotypes, the majority of the NC-CAH patients (Group I) were homozygous for the p.Val282Leu pathogenic variant (58% of the children and 50% of the adults) or compound heterozygous for the p.Val282Leu/del/conv promotor *CYP21A2* pathogenic variants (17% of the children and 50% of the adults). As for the heterozygous carriers (Group II), the majority were simple heterozygous for the p.Val282Leu variant (90% of the children and 100% of the adults).

Clinical features did not significantly differ between patients homozygous for the p.Val282Leu variant and patients with the p.Val282Leu/del/conv genotype. Median basal and stimulated 17OHP were higher in homozygous than in compound heterozygous patients (respectively, 12.3 *vs.* 6.9 ng/mL (p = 0.367), and 17.8 *vs.* 19.0 ng/mL (p = 0.141)).

## DISCUSSION

We have analyzed a population of 131 females suspected of having NC-CAH due to clinical and biochemical criteria, including 46 NC-CAH individuals, 49 heterozygous carriers, and 36 wild type subjects. We have explored our data in two groups (32 children and 99 adolescents and women), as we realize that young girls with premature pubarche are clinically different from adolescents and women with hirsutism, oligomenorrhea, or infertility. However, we found similar results regardless of the age group, including similar signs and symptoms and various 17OHP levels between groups.

Heterozygous carriers presented higher basal and ACTH-stimulated 17OHP levels than wild type subjects and lower basal and stimulated 17OHP levels than NC-CAH patients. In accordance with our data, some previous studies showed higher basal and/or stimulated 17OHP levels in heterozygous *CYP21A2* pathogenic variant carriers than in non-carriers ( 5 , 9 , 10 , 16 , 17 , 25 – 28 ). Other studies, though, showed no significant differences in hormone levels between heterozygous carriers and the general population ( 12 , 14 , 18 , 19 ). Conflicting results may have arisen due to differences in the participants’ selection criteria.

In our population, 17OHP concentrations exhibit significant overlap between carriers and the remaining two groups. In heterozygous carriers and wild type individuals, we found post-ACTH 17OHP levels > 3 ng/mL, a cutoff proposed for the identification of carrier *status* ( 28 , 29 ), and even some > 10 ng/mL, the cutoff usually applied for the NC-CAH diagnosis ( 30 ). Therefore, we state that these groups cannot be distinctly separated according to biochemical criteria and underline that the detection of the carrier *status* should be based on molecular genotype analysis.

Admoni and cols. distinguished two groups of carriers, a group of symptomatic carriers presenting with premature pubarche, accelerated growth, and hirsutism and a group of asymptomatic family member carriers, and they stated that the symptomatic carriers had higher cosyntropin-stimulated 17OHP levels and a higher rate of the p.Val282Leu pathogenic variant than family member carriers ( 27 ). They suggested that the subjects who carried the mild p.Val282Leu pathogenic variant had higher rates of androgen excess symptoms than the carriers of other severe pathogenic variants and speculated that the impairment of enzymatic activity in the symptomatic carriers results from this mutant allele’s dominant-negative effect on the wild type in which the mutant enzyme may compete or interfere with the wild type for the conversion of 17OHP to 11-deoxycortysol ( 27 , 31 ). The monoallelic pathogenic variant affects the normal allele’s function, leading to a slight reduction in the 21OH activity, increased androgens secretion, and subsequent clinical manifestations ( 31 ). Other studies have supported this observation and showed higher rates of either PCOS or hirsutism in p.Val282Leu carriers but not in other mutation carriers ( 9 , 27 , 32 ). In our population, the large majority of our carriers were simple heterozygous for the p.Val282Leu variant (90% of the children and 100% of the adults), which is the most frequent variant in the Portuguese population ( 3 ). This fact may explain our carriers’ higher 17OHP levels compared to wild type subjects, and we may question whether we would see different results if our heterozygous subjects carried other pathogenic variants.

Possible explanations for the phenotypic heterogeneity among individuals carrying the same pathogenic variant include the activity of other genes encoding proteins with extra-adrenal 21OH activity, the presence of other still unidentified mutations, the activity of other genes encoding proteins with extra-adrenal 21OH activity, and interindividual variation in androgens’ peripheral sensitivity and in the amount of protein produced ( 33 ).

In our population, we found no significant differences in signs and symptoms between NC-CAH patients, heterozygous carriers, and wild type individuals. We only included females in the genetic study for the *CYP21A2* gene and selected them based on their hormonal patterns and clinical features; therefore, the similarities between the three groups did not surprise us. Moreover, we obtained our data from clinical records, which we must interpret carefully. Our hyperandrogenic wild type girls probably presented premature adrenarche, a diagnosis of exclusion, while most of our post-menarche woman presented polycystic ovarian syndrome (PCOS). Premature adrenarche and PCOS are difficult to differentiate from NC-CAH and heterozygous carrier *status* based on clinical findings. We recommend treatment of symptomatic heterozygote carriers for the symptoms of androgen excess with both pharmacological therapies, including glucocorticoids and cosmetic approaches ( 29 ).

It would have been interesting to compare 17OHP levels in individuals showing one hyperandrogenic sign or symptom *versus* individuals showing three or more hyperandrogenic signs or symptoms, both in the wild type and in the carriers group. However, due to this study’s retrospective nature, we could not conduct this analysis.

Limitations include the small sample size, our study’s retrospective design, and the fact that heterozygous carriers with monoallelic pathogenic variants were not studied for other point sequence variants within regulatory or epigenetic control regions in the CYP21 locus, mainly the analysis of the 3’untranslated region of the *CYP21A2* gene (3′UTR region), where it was recently reported that 3’UTR sequence variants in combination with other pathogenic variants may be associated with a mild form of the disease ( 34 ). More accurate biochemical predictors of the heterozygous carrier status, such as the serum 21-deoxycortisol ( 35 , 36 ) or the 17OHP/cortisol ratio ( 37 ), were not available in our population. We did not analyze data regarding 17OHP levels in women without hyperandrogenic manifestations.

In conclusion, we found no significant differences in clinical features between NC-CAH patients, heterozygous carriers, and wild type females. The most common pathogenic variant in the *CYP21A2* gene identified among NC-CAH patients and carriers was p.Val282Leu. Heterozygous carriers presented higher basal and post-cosyntropin 17OHP levels than wild type individuals and lower basal and stimulated 17OHP levels than NC-CAH patients. We discovered a considerable overlap between 17OHP levels among groups; therefore, normal basal and stimulated 17OHP responses do not exclude heterozygosity for *CYP21A2* pathogenic variants.
